# Acute Severe Asthma in Adolescent and Adult Patients: Current Perspectives on Assessment and Management

**DOI:** 10.3390/jcm8091283

**Published:** 2019-08-22

**Authors:** Eirini Kostakou, Evangelos Kaniaris, Effrosyni Filiou, Ioannis Vasileiadis, Paraskevi Katsaounou, Eleni Tzortzaki, Nikolaos Koulouris, Antonia Koutsoukou, Nikoletta Rovina

**Affiliations:** 1 ICU, 1st Department of Pulmonary Medicine, “Sotiria” Hospital, Athens School of Medicine, National and Kapodistrian University of Athens, 11527 Athens, Greece; 2 1st ICU, Evangelismos Hospital, Athens School of Medicine, National and Kapodistrian University of Athens, 11527 Athens, Greece; 3 Respiratory Outpatient Clinic, 71305 Heraklion, Greece

**Keywords:** acute severe asthma exacerbation, near fatal asthma

## Abstract

Asthma is a chronic airway inflammatory disease that is associated with variable expiratory flow, variable respiratory symptoms, and exacerbations which sometimes require hospitalization or may be fatal. It is not only patients with severe and poorly controlled asthma that are at risk for an acute severe exacerbation, but this has also been observed in patients with otherwise mild or moderate asthma. This review discusses current aspects on the pathogenesis and pathophysiology of acute severe asthma exacerbations and provides the current perspectives on the management of acute severe asthma attacks in the emergency department and the intensive care unit.

## 1. Introduction

Asthma is a chronic inflammatory disorder of the airways, a common and potentially serious chronic disease that is associated with variable expiratory flow, airway wall thickening, respiratory symptoms, and exacerbations (flare-ups), which sometimes require hospitalization and may be fatal [1]. In reference to asthma, an exacerbation is defined as an event characterized by change from the patient’s previous status, including a progressive increase in relevant symptoms and a decrease in respiratory function. The latter can be quantified by respiratory function measurements such as peak expiratory flow (PEF), and forced expiratory volume in 1 s (FEV_1_), which when compared with the patient’s previous or predicted values, reflect the deterioration in expiratory airflow, the prominent pathophysiological effect of an asthma attack.

The most common causes of these exacerbations are exposure to external agents, such as indoor and outdoor allergens [2,3,4], air pollutants [5], and respiratory tract infections (primarily viral mainly human rhinovirus (HRV) [6,7]. The mechanisms by which these environmental stimuli and viruses initiate asthma or cause worsening of the disease are under research.

Asthma exacerbations may also be triggered by exercise [8], weather changes [9], foods [10,11], additives, drugs [1,2,3,4,5,6,7,8,9,10,11,12,13,14], and extreme emotional expressions [15,16]. The physiological hallmarks of asthma are airway inflammation, airway remodeling and bronchial hyperresponsiveness (BHR) [17]. Exposure to the above-mentioned external stimuli and specifically to inhaled allergens is capable of inducing an inflammatory response in sensitized individuals and as a result to lead to exacerbations [18,19]. A hypothesis explaining this fact is that the inflammatory response resulting from inhaled allergen may drive BHR directly, or induce structural changes in the airway leading to persistent BHR [17,20]. Experimental mouse models of asthma have shown that allergen exposure protocols induce immune-mediated airway inflammation defined by: elevated levels of asthma biomarkers (IgE, the T-helper cell 2 (Th2) cytokines, interleukins (IL)-4, -5 and -13, and eosinophils), induction of airway remodeling (increases in airway smooth muscle, collagen deposition and goblet cell hyperplasia), and BHR that is sustained after the resolution of eosinophilic inflammation [21,22,23].

Other factors that may cause exacerbations are rhinitis [24] or sinusitis [25], polyposis [26], gastroesophageal reflux [27], menstruation [28,29], or even pregnancy [30,31]. They can happen either to patients with known asthma of any level of severity, or less frequently as a first presentation. Exacerbations vary in severity, as well as in response to therapy. This has led to an effort of categorize the severity of these exacerbations. The most frequently proposed categories include elements of the clinical presentation of the asthma patient, as well as a measurement of their respiratory function at the time of the exacerbation. It is of paramount importance for the clinician to distinguish the severe exacerbations, because these are the ones that correlate with worse consequences.

## 2. Definition of Acute Severe Asthma

The Global Initiative for Asthma guidelines refers to a severe asthma exacerbation describing a patient who talks in words, leans forward, is agitated, uses accessory respiratory muscles, has a respiratory rate > 30/min, heart rate > 120/min, O_2_ saturation on air < 90% and PEF ≤ 50% of their best or predicted value [1]. According to the 2014 British Guidelines for Asthma, acute severe asthma is defined as the asthma exacerbation that presents with any of the following: PEF 33–50% best or predicted, respiratory rate ≥ 25/min, heart rate ≥ 110/min and inability to complete sentences in one breath [32]. The ATS/ERS task force defines a severe asthma exacerbation by the fact that they require urgent action in order to prevent a serious outcome, such as hospitalization or death from asthma [33]. This task force recommends that the definition of a severe asthma exacerbation for clinical trials should include at least one of the following: (a) use of systemic corticosteroids (tablets, suspension, or injection), or an increase from a stable maintenance dose, for at least three days; and (b) a hospitalization or emergency department visit because of asthma, requiring systemic corticosteroids. Although these definitions are not identical, the point remains that identifying this condition is important as it is correlated with worse outcomes and greater risk of needing mechanical ventilation.

There are other entities similar but not identical to that of acute severe asthma that also require precise definitions. Kenyon et al. proposed the term Critical Asthma Syndromes (CAS) to identify any child or adult who is at risk of fatal asthma [34]. This term includes acute severe asthma, refractory asthma, status asthmaticus, and near fatal asthma, all of them conditions that can lead to respiratory exhaustion and arrest. Refractory asthma is, according to a definition set by the Unbiased Biomarkers for the Prediction of Respiratory Disease Outcomes (U-BIOPRED) consortium in 2011, patients with asthma in whom after excluding any alternative diagnoses, after treating comorbidities and removing trigger factors cannot maintain good asthma control, despite high-intensity treatment and confirmed compliance with treatment. These patients have frequent severe exacerbations (≥2 per year), or can only be well when receiving systemic corticosteroids [35]. Near fatal asthma (NFA) is defined as an asthma exacerbation resulting in respiratory arrest requiring mechanical ventilation or a pCO_2_ ≥ 45 mm Hg. Some writers tend to recognize status asthmaticus and acute severe asthma as the same condition and define it mainly by its response to treatment, thus referring to it as an exacerbation that does not respond to repeated courses of β2-agonist therapy [36].

## 3. Epidemiology

**Figure 1:**
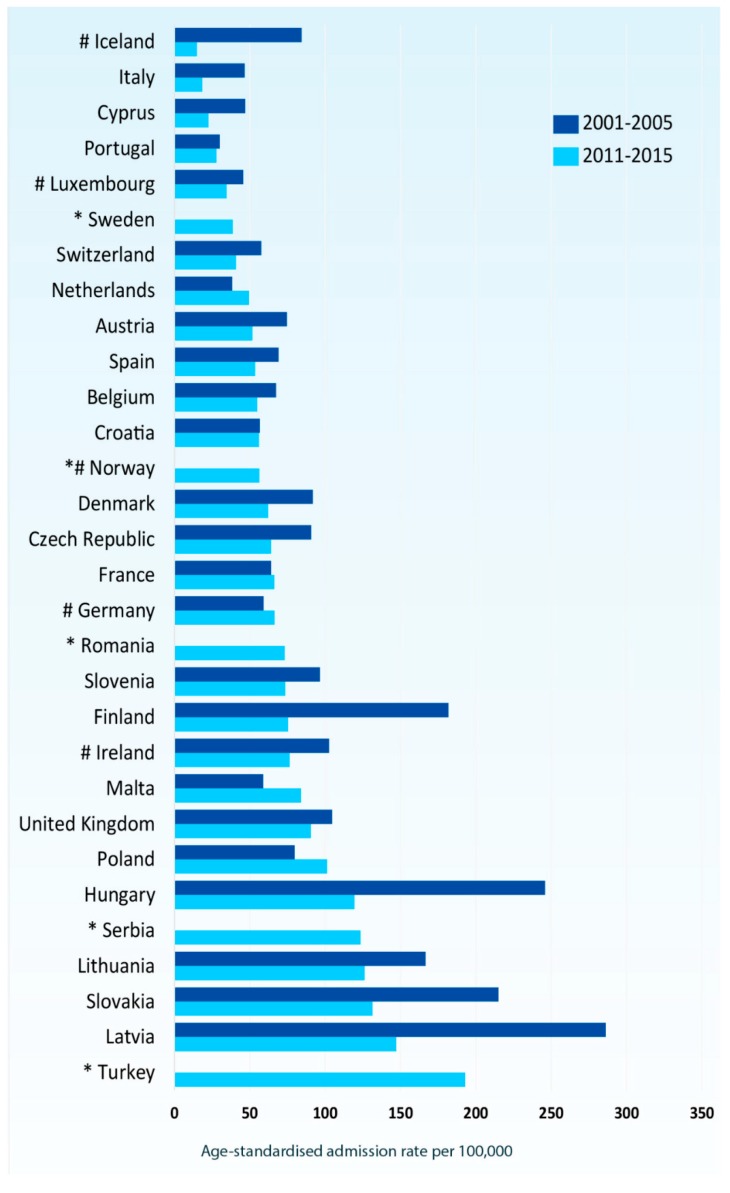
Age-standardized admission rates for asthma (all ages) in 30 European countries in two time periods: 2001–2005 and 2011–2015. Source: Eurostat updated from ec.europa.eu/Eurostat/web/health/health-care/data/database (version dated November 2017).

**Figure 2:**
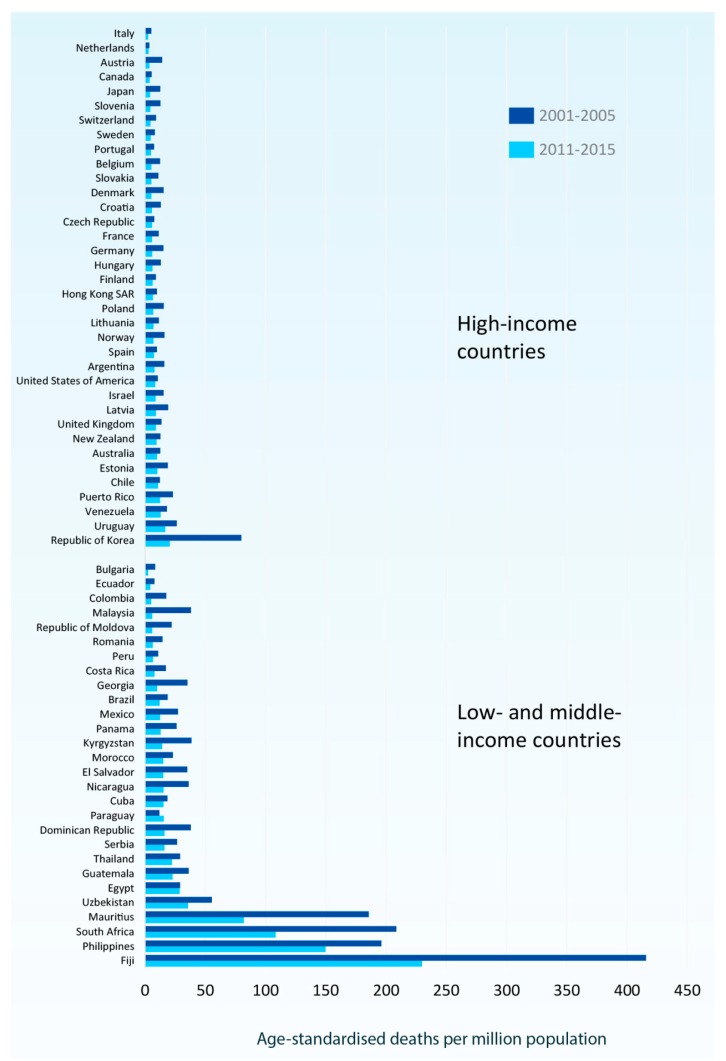
Age-standardized mortality rates for asthma (all ages) by country in two time periods: 2001–2005 and 2011–2015. Source: Eurostat updated from ec.europa.eu/Eurostat/web/health/health-care/data/database (version dated November 2017).

According to the Global Asthma Report, approximately 334 million people in the world suffer from asthma, thus being the most prevalent chronic respiratory disease, with chronic obstructive pulmonary disease (COPD) affecting only half of the aforementioned number of people [37]. However, according to Eurostat [38], in most European countries age standardized asthma admission rates declined from 2001–2005 to 2011–2015, with an over two-fold reduction in some countries. (Figure 1) The latest World Health Organization (WHO) estimates, released in December 2016, present that there were 383,000 deaths due to asthma in 2015. There has been a decrease of almost 26% in the asthma deaths, when comparing 2015 to 1990 [37]. However, international mortality statistics for asthma are limited to those countries reporting detailed causes of death. Figure 2 depicts the age-standardized mortality rates for asthma among countries reporting asthma separately in two recent five-year periods (2001–2005 and 2011–2015) [38].

Although asthma is a disease not only of low- and lower-middle-income countries, most asthma-related deaths occur in those areas [38]. There is an established connection between asthma deaths and the Socio-Demographic Index (SDI), but interestingly not with SDI and asthma prevalence. A recent study in Brazil demonstrated that urbanization has affected public health, resulting in higher asthma related morbidity and mortality, despite the fact that the urbanized population now has improved access to the health system [39]. There are no accurate figures describing the rate of acute severe asthma, but there are sufficient data regarding the asthma related hospitalizations and asthma related mortality. Recent studies estimate the risk of death of the patients who are hospitalized as a result of asthma exacerbation as less than 0.5% [40,41]. That risk is greater when the patient requires intubation and mechanical ventilation, which underlines the importance of identifying and promptly treating acute severe asthma. Four percent of asthma related hospitalizations result in mechanical ventilation. There is a substantial economic burden associated with asthma hospitalizations, and it has been demonstrated that in the US in 2012 the overall cost was more than 2 billion dollars, which is a significant percentage (more than 1/3) of the annual asthma related expenditure [41]. Middle aged women are more likely to get hospitalized with asthma related morbidities [41].

With regards to the identified phenotypes of asthma, data from a recent cluster analysis from Japan revealed a wide heterogeneity among asthma patients who presented and were admitted with severe and life-threatening asthma in 17 institutions across the country [42]. Another recent group-based trajectory analysis on patients with problematic and uncontrolled asthma, showed that near fatal events were noted in all groups, but were more frequent in patients with persistent frequent exacerbations [43]. It is not only patients with severe and poorly controlled asthma who are at risk for having an acute severe asthma exacerbation, but this has been observed as well in patients with otherwise mild or moderate asthma. The current literature describes two distinct clinico-pathophysiological entities of acute severe asthma attacks that present at the emergency department: the slow onset, late arrival and the sudden onset fatal asthma. It has been estimated that the majority (80–85%) of asthma-related fatalities belong to the slow onset group. These patients may have symptoms and uncontrolled disease for several days prior to the presentation with acute severe asthma. Sudden onset has been defined as severe airflow obstruction established after 1–3 h of symptom presentation. Barr et al. reported that patients presenting with sudden onset asthma, were more likely to have been exposed to an exacerbation trigger such as a respiratory allergen, exercise and psychosocial stress and less often respiratory infection and had greater improvement when compared with the slow onset cohort [44]. A retrospective cohort study in the United States demonstrated evidence that the sudden-onset patients were older, were more likely to present during the night and early morning hours at the emergency department, more often required intubation and mechanical ventilation, and had higher rate of ICU admission, but, on the other hand, had shorter hospital stay [45]. In this study the sudden onset cohort was only 6% of 1260 patients in 30 hospitals.

## 4. Risk Factors for Asthma Exacerbations

Many factors have been studied regarding their correlation with acute severe asthma and asthma related death (Table 1). In adults, asthma exacerbations are more often in females [46,47]. This is difficult to be explained since female asthmatics have lower levels of total serum IgE [48] and the incidence of atopy is actually lower in comparison to males [49]. A possible explanation could have to do with the connection between asthma worsening and the menses, which is a recognized contributing factor of asthma worsening [50]. Furthermore, pregnancy in asthmatic women is a condition that requires special considerations, considering the effect of the disease, as well as the medication on the mother and the fetus. Pregnancy is not always correlated with worse asthma control, although there seems to be a correlation between asthma severity and morbidities and exacerbations during pregnancy [51]. There has been reported a cluster of obese females with late-onset corticosteroid asthma with frequent exacerbations although they preserve a relatively good baseline lung function [52].

Obesity per se has also been correlated with worse asthma control, as well as more frequent and severe exacerbations. This correlation is strengthened by the apparent effect of weight loss and bariatric surgery on better control and less exacerbations and hospitalizations [53].

Ethnicity and socioeconomic status [54,55] are robust determinants of asthma exacerbation rates. African Americans have 4.2- and 2.8-fold higher rates of emergency room visits and hospitalizations for asthma exacerbation, respectively, compared to Caucasians, followed by Hispanics [39]. A possible explanation for these differences could be the poorer adherence to treatment [56] and the poorer quality of healthcare in ethnic minorities [57]. A significant genetic component might also contribute, since an increased risk of exacerbations has been documented in males with African ancestry [58].

Severe exacerbations may occur in patients with mild or well controlled asthma [59,60]. However, poor asthma control is an independent risk factor for future acute exacerbations [61,62,63,64,65]. A history of a recent exacerbation is the strongest predictor of future exacerbations in children and adults with asthma [66,67,68,69]. A small percentage of asthmatics exhibit severe disease exacerbations, despite the fact that they are already under treatment with high doses of inhaled and/or systemic corticosteroids [70,71]. These patients suffering from severe asthma (SA) that is poorly controlled and in some cases life-threatening [34,35], although comprising a small percentage of the total asthma population (5–10%), they denote 50% of total healthcare costs, rendering SA a substantial health and socio-economic burden [36,37].

Finally, poor perception of airflow limitation may affect patients with a history of near-fatal asthma and appears to be more common in males [72,73]. On the other hand, regular or overuse of short acting beta agonists (SABA) causes down regulation of beta receptors and leads to lack of response, leading in turn to overuse [74]. Overuse may also be habitual. Dispensing ≥3 SABA canisters/year (average 1.5 puffs/day or more) is associated with increased risk of emergency department visits or hospitalizations no matter what the severity of asthma is [75], while dispensing ≥12 canisters/year (1/month) increases the risk of death [76]. Incorrect inhaler technique (seen in up to 80% of asthma patients) [77], as well as suboptimal adherence to treatment (seen in up to 75% of patients) are important modifiable factors contributing to symptoms and exacerbations [77].

There has been a lot of interest regarding the effect of psychological factors on the risk for fatal or near fatal asthma, this however has not been established, as shown in a 2007 systematic review by Alvarez et al. [78]. Anxiety, depression and socio-economic problems are very common in patients with difficult to treat asthma and contribute to poor symptom control, poor adherence to treatment and impaired quality of life [79].

Obesity and other comorbidities other than the psychiatric conditions already mentioned that contribute to persistent symptoms, exacerbations and poor quality of life include chronic rhinosinusistis [80], inducible laryngeal obstruction (often referred as vocal cord dysfunction, VCD), gastroesophageal regurgitation disorder (GERD), chronic obstructive pulmonary disease (COPD), obstructive sleep apnea, bronchiectasis, cardiac disease, and kyphosis due to osteoporosis (followed by corticosteroid overuse) [80].

## 5. Factors that Trigger Asthma Exacerbations

Severe exacerbations usually occur in response to a variety of external agents (e.g., respiratory pathogens, allergens, air pollutants, smoke, and cold or dry air).

### 5.1. Respiratory Pathogens

Viral respiratory infections are the most common triggers for a severe asthma exacerbation, comprising up to 76–80% of the causes of an acute asthma exacerbation in adults [81]. Human rhinovirus (RV) (types A and C), influenza virus (types A and B), para-influenza virus, and respiratory syncytial virus (RSV) are frequent causes of an acute exacerbation and hospitalization [56,82]. Coronaviruses, meta-pneumoviruses, bocaviruses, and adenoviruses may also trigger a severe acute exacerbation, however to a lesser extent [57]. During the 2009 H1N1 influenza A pandemic, mortality and admissions to the ICU with H1N1 infections were frequently associated with asthma [82,83]. In contrast to other respiratory viruses (i.e., RSV and Influenza Virus), RV does not exert a definite cytopathic effect [84]; instead, it compromises the function of the epithelial barrier through the release of reactive oxygen species during viral replication [85]. During this process, the induction of immune and adaptive immune response activates the synthesis and early secretion of IFNs and other pro-inflammatory cytokines (i.e., IL-10, IL-6, IL-8, RANTES, and ENA-78) [86], which play a significant role in the protective mechanisms against viral infection [87,88]. There is evidence that in asthmatic patients there is dysregulated immune response against RV [89]. Several studies have demonstrated the implication of interferons in the susceptibility to asthma exacerbations in children and adults in the context of a viral respiratory infection. Miller et al. [90] showed that RV was related to asthma exacerbation with the implication of IFN III. Similarly, Jones et al. [91] documented an increased susceptibility to severe respiratory viral infections during the first years of life through dysregulated type III IFN responses, while recent studies [92,93] document a varying susceptibility to asthma exacerbations depending on the type and level of expression of cytokines and IFNs upon viral infection. Finally, Fedele et al. [94] documented that RV infection more frequently induces a Th2-mediated immune response than RSV infection, justifying the higher incidence of asthma prevalence after RV infections.

Bacterial infections may also trigger acute exacerbations, usually on the basis of impaired anti-bacterial defense after a viral respiratory infection [95]. There are bidirectional interactions between viruses and bacteria that seem to have an impact on the severity of asthma as well as the likelihood of an acute exacerbation. Viral infections facilitate the disruption of airway epithelial layers and the expression of airway receptors that bacteria use in order to invade [96]. Furthermore, in the presence of co-infection, an increased release of inflammatory cytokines and mediators is induced, heightening the burden of inflammation and predisposing to a higher risk of exacerbations [97]. Specifically, co-infections of respiratory viruses and *Moraxella catarrhalis*, *Hemophilus influenza*, and/or *Streptococcus pneumonia* have a greater impact on the risk for more severe acute asthma exacerbations [97]. The clarification of the mechanisms implicate the case of co-infections on inter-relationship for providing evidence for potential novel therapeutic targets that may prevent acute asthma exacerbations.

### 5.2. Allergen Sensitization and Viral Infections

Evidence support the theory that allergic sensitization increases the susceptibility for viral infections and probably their ability to provoke further inflammation [98].

For example, it has been shown that the combination of RV infection and direct exposure to allergens cause epithelial cell production of IL-25 and IL-33 in the airways, mediators involved in Th2 type inflammation and remodeling [99,100]. Moreover, in a murine model of asthma RV infection acquired in early life stages in mice induced an IL-13- and IL-25-mediated Th2 immune response with parallel suppression of IFN-γ, IL-12, and TNF-α [101], with detrimental changes in airway homeostasis, consisting of innate lymphoid cell expansion, mucous hypersecretion, and airway responsiveness. Furthermore, recurrent RV infections stimulate airway remodeling by upregulating molecules such as VEGF and TGF-β, as well as chemoattractants for airway smooth muscles (i.e., CCL5, CXCL8, and CXCL10) [102,103].

Other data show that the occupancy of the IgE membrane receptors inhibits antiviral induction of interferon-a from plasmacytoid dendritic cells leading to subsequent increased susceptibility to viral infections and asthma exacerbations. It is noteworthy that an inverse correlation between interferon levels and airway eosinophilia, IL-4 levels, and total serum IgE was observed [104].

### 5.3. Allergen Exposure, Tobacco Smoke, and Environmental Pollutants

Indoor or outdoor exposure to allergens may lead to poor asthma control and severe asthma exacerbations in sensitized patients [105,106,107,108,109]. Allergens activate mast cells to release histamine, prostaglandin D2, and cysteinyl leukotrienes. These induce inflammatory responses, airway smooth muscle constriction, increased microvascular permeability, and mucus secretion, diminishing at the same time the innate immune responses and subsequently increasing the susceptibility to viral infections [106,107]. Of great importance is the mold sensitization, which has been associated with the phenotype of severe asthma and with severe asthma attacks. High airborne concentrations of mold have been associated with increased emergency visits for asthma exacerbations [108]. Specifically, Alternaria is associated with highly increased risk (almost 200-fold) of severe exacerbations and need for ICU admittance in both children and adults [109]. Furthermore, cockroach and mouse antigens are associated with early wheeze and atopy in an inner-city birth cohort [110].

Exposure to multiple allergens has been documented as being a common feature in several studies of indoor exposure [111,112]. Salo et al. [112] showed that more than 50% of subjects were sensitized at least to six detectable allergens, while 45% were sensitized at least to three allergens. In a study from China, Kim et al. [111] showed sensitization to one or more allergens in almost 50% of the subjects with most common sensitizers being shellfish, dust mites, and cockroaches. However, less than 1% of these subjects had clinically important food allergy or asthma.

Indoor exposure to endotoxin and pollutants (such as particulate matter and nitrogen dioxide) has also been found to increase the risk of severe exacerbations in children with asthma and the use of particulate filters seem effective in reducing exposure levels and therefore, asthma control [113,114]. Differences in allergic sensitizations by race and genetic ancestry have also been documented [115], and along with the location of residence seem to be more important predictors of allergic sensitization than genetic ancestry. This fact points out the hypothesis that disparities in allergic sensitization by race may be observed as an effect of environmental rather than genetic factors.

Tobacco smoke remains one of the most significant triggers of disease, despite increased public awareness of the detrimental effects of smoking. Asthma patients who smoke have more frequent emergency department visits and hospitalizations for an exacerbation than asthma patients who do not smoke [116]. Several studies of patients with allergic rhinitis have shown the significant effect of smoking on the development of asthma. Polosa et al. [117] showed that in a 10-year period smoking had a dose-related effect on the development of asthma in allergic rhinitic patients resulting in an odds ratio of 2.05 for incident asthma for smoking 10 pack-years, and 3.7 and 5.05 for 11–20 and >20 pack-years, respectively.

Second-hand smoke is also associated with deteriorated lung function, poor treatment response, and frequent emergency department visits for asthma [118,119,120]. The measurement and monitoring of cotinine levels in serum, urine, and saliva have become a useful tool in determining passive smoke exposure as well as in evaluating uncontrolled asthma. Hassanzad et al. demonstrated that higher cotinine levels were associated with a higher risk for severe asthma. [121]. Increasing interest has also raised on the potential hazards of third-hand smoke (THS) in children. THS is residual nicotine and other chemical pollutants remaining in the indoor environment and on household surfaces for weeks to months after active tobacco smoking has stopped. It seems that young children may be more susceptible to the adverse effects of THS exposure since they crawl and tend to ingest several items from the surrounding [122]. However, more research is needed to assess the real extent of the hazards arising from THS.

Environmental pollutants, such as particulate matter, ozone, sulfur dioxide, nitrogen dioxide, and diesel exhaust, may act synergistically with viral infections to cause asthma exacerbations [123] The effects of air pollution on severe asthma exacerbations may be affected by other exposures, such as stress, vitamin D insufficiency, and seasonality [4,5]. This was demonstrated in a study of children aged 0–18 years in California, where particulate matter (size, 2.5 mm; PM_2.5_) and ozone were associated with severe asthma exacerbations in the warm season, while in the cool season exacerbations were associated with articulate matter of PM_2.5_, carbon monoxide, and NOx (NO_1_NO_2_) [124,125].

## 6. Genetic Associations with Asthma Exacerbations

Genome-wide association studies of asthma in children and adults have identified polymorphisms for IL33, IL1RL1/IL18R1, HLA-DQ, SMAD3, and IL2RB9 and the locus on chromosome 17q21 including the genes ZPBP2, GSDMB, and ORMDL3 that are implicated in epithelial barrier function and innate and adaptive immune responses in asthma [126,127]. Genetic variants in the class I major histocompatibility complex-restricted T cell-associated molecule gene (CRTAM) was associated with an increased rate of asthma exacerbations in children with low circulating vitamin D levels [128]. One of the most well replicated genetic regions affecting asthma risk is the 17q12–21 locus, which includes ORMDL3 and GSDMB. The TT allele at rs7216389 is associated with an odds ratio of 1.6 of having an asthma exacerbation when compared with the CC allele [129].

Furthermore, polymorphisms for FCER2 have been associated with decreased FCER2 gene expression, increased serum IgE levels and risk of severe exacerbations [130]. Association was also found between variants in chitinase 3-like 1 (CHI3L1; YKL-40) and asthma exacerbations and hospitalizations [131,132]. Specifically, studies in murine models of asthma implicate YKL-40 in IgE induction, antigen sensitization, dendritic cell accumulation and activation, and alternative macrophage activation [133], while purified YKL-40 induces interleukin-8 secretion in bronchial epithelial cells [134].

## 7. Pathogenesis-Immunobiology

**Figure 3:**
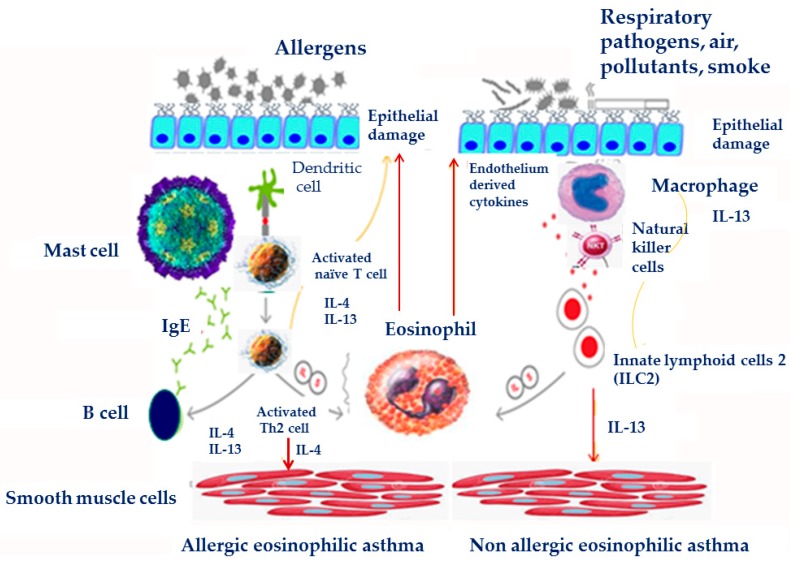
Pathogenesis of acute exacerbations in asthma.

Asthma is a heterogeneous condition with complex observable characteristics (phenotype) and their underlying mechanisms (endotype), resulting from complex host–environment interactions (Figure 3). Usually, inflammatory cells are present and activated in the airways of severe asthmatics and persist despite treatment, but their relevance to lack of control and disease severity is largely unknown. These cells include not only eosinophils and neutrophils, but also T-lymphocytes, mast cells, macrophages and airway structural cells which are also crucially involved in the inflammatory reaction and remodeling in asthma. Although it is well accepted that asthma is characterized by eosinophilic infiltration, inflammatory phenotypes of severe asthma can be characterized by persistence of eosinophilic or neutrophilic infiltration, as well as by absence of inflammatory infiltration (paucigranulocytic) [135,136]. Depending on the type of immune cell responses implicated in disease pathogenesis, asthma endotypes are categorized as type 2 asthma, characterized predominantly by T helper type 2 (Th2) cell-mediated inflammation and non-type 2 asthma, associated with Th1 and/or Th17 cell inflammation [137,138]. Eosinophilic, Th2 airway inflammation is present in around 50% of adults with asthma, and is estimated to be higher in the absence of corticosteroids [139].

Th2 mediated airway inflammation plays a central role in the pathophysiology of allergic eosinophilic asthma. The allergic sensitization of dendritic cells (DCs) in the presence of thymic stromal lymphopoietin (TSLP), induces Th2 lymphocytes to produce cytokines such as interleukins IL-4, IL-5, and IL-13 [140]. Chemokines such as eotaxin 1, 2, 3 (CCL11, CCL24 and CCL26, respectively) induce through their receptors (chemokine receptor 3, CCR3) [141] and other chemoattractant agents, such as mast cell derived prostaglandin D2 (PGD2) eosinophil recruitment in the mucosa. Furthermore, IL-4 and IL-13 activate B lymphocytes to produce allergen specific IgE, which binds to the high affinity mast cell receptors, leading to their activation [140].

In non-allergic eosinophilic asthma, airway epithelial damage caused by pollution and pathogens leads to IL-5 and IL-13 production by innate lymphoid cells (ILC2s), in response to PGD2, TSLP, IL-25 and IL-33 [142]. ILC2s and Th2 cells are a significant source of type 2 cytokines and play a role in eosinophilic inflammatory response, allergy and remodeling in asthma [143,144]. Increased circulating and sputum IL-5 and IL-13-producing ILC2s were detected in severe asthma compared to mild asthma patients [145]. Furthermore, increased numbers of IL-5^+^ and IL-13^+^ ILC2s were found in sputum after allergen challenge in asthma patients [146]. IL-13-expressing ILC2 and Th2 cells are also responsible for bronchial epithelial tight junction barrier leakiness in asthma patients [147,148].

Chronic inflammation that characterizes severe asthma leads to tissue remodeling, fixed airway obstruction, and no response to bronchodilatory treatment [149]. It seems that chronic persistent inflammation and the release of a plethora of cytokines (IL-5, IL-9, IL-13, osteopontin, and activin-A9), chemokines (CCR3 dependent) and growth factors (TGF-β1 and VEGF) from inflammatory and epithelial cells play a central role in the establishment of airway remodeling [150].

Physiologically, airway inflammation is counteracted by inhibitory molecules and suppressor cells including CD4^+^ regulatory T cells (Tregs) [151,152] which becomes visible upon Treg depletion which causes spontaneous asthma-like airway pathology [153]. Patients suffering from allergic asthma have reduced numbers of Tregs that furthermore show impaired suppressive capacity [154,155,156,157]. Some currently applied therapies aim at enhancing Treg cell number and function [154,158], whereas adoptive transfer of Tregs can suppress both the priming and the effector phase of allergic airway inflammation in experimental models of murine asthma [159,160,161].

Mixed eosinophilic and neutrophilic inflammation of the airways are commonly found in severe asthma [162] and this mixed inflammatory pattern can be a biomarker of the most severe types of the disease [163]. Elevated sputum neutrophil counts were found to be associated with more severe asthma phenotypes and with poor response to treatment with steroids in a cluster analysis from the Severe Asthma Research Program (SARP) [164]. Airway neutrophilia has been associated with persistent airflow obstruction in patients with refractory asthma and a progressive loss of lung function [165] Furthermore, it is associated with higher bronchial hyperresponsiveness independent of eosinophilia [166].

It is suggested that increased neutrophil counts in peripheral blood and sputum could be secondary to the treatment with corticosteroids, since the anti-apoptotic effect of corticosteroids on neutrophils is well established [167]. However, neutrophilic inflammation may be observed regardless of corticosteroid treatment in patients with refractory asthma or in patients experiencing acute severe exacerbations [168,169,170].

**Figure 4:**
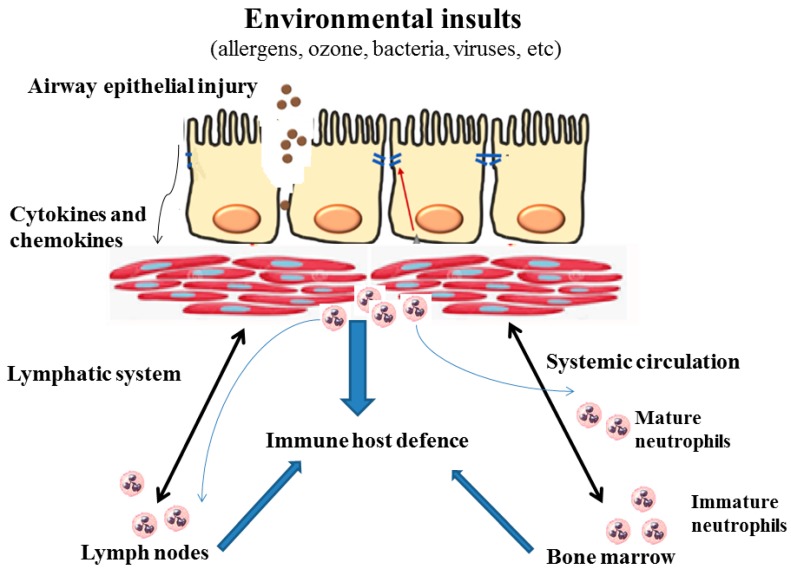
The role of the neutrophil in modulating local inflammatory responses.

Neutrophil recruitment and activation into the airways have been associated with stimulation of toll-like receptors (TLR) signaling and activation of innate immunity, causing a shift toward Th1 and Th17 responses. This process leads to increased production of interleukin (IL)-8, IL-17A, neutrophil elastase, and matrix metalloproteinase 9 [171]. Neutrophils are triggered by IL-8 to produce enzymes and other factors that contribute to eosinophil activity [171]. Evidence suggests that neutrophil subsets may mediate differential effects on immune surveillance and microbial killing. A variety of epithelial insults (ozone, bacteria, and viruses) induce secretion of chemokines and cytokines that promote neutrophil trafficking. Neutrophils primarily traffic to inflamed sites and then secrete granular enzymes, reactive oxygen species, and proteins to eliminate invading bacteria, fungal elements, and viruses. Undoubtedly, neutrophils play pivotal roles in innate immunity [172]. During asthma exacerbations, the presence of chemokines and cytokines (IL-8 and IL-17A) prolongs neutrophils’ lifespan thus enabling them to migrate from tissue to the systemic circulation or to lymph nodes to modulate adaptive immune responses, Figure 4. The combined functions of these cytokines and activated enzymes promote airway structures to contribute to the lower FEV_1_, remodeling and fixed airway obstruction seen in adult patients with severe neutrophilic asthma [173].

## 8. Biomarkers Correlating with Risk of Asthma Exacerbations

The better understanding of the pathophysiology of asthma has led to the recognition of biomarkers with a potential to predict severe exacerbations. Among T2-high asthma biomarkers sputum and blood eosinophil count, serum IgE, serum periostin levels, and levels of nitric oxide in exhaled breath (FeNO) seem to associate with the severity of asthma and the rate and severity of exacerbations.

Sputum eosinophils have been correlated with increased asthma severity and airway responsiveness. Increased sputum eosinophil counts have been used as a measure of better response to corticosteroid treatment, in terms of reducing exacerbations. In the systematic review by Petsky et al. [174] it was demonstrated that asthma treatment guided by sputum eosinophil counts led to a significant reduction in the exacerbation rate. In children, elevated blood eosinophil count is associated with persistent asthma symptoms, and responsiveness to treatment can be predicted by the number of eosinophils without having set though a validated cut-off point [175]

Baseline blood eosinophil count is being used as a biomarker that predicts the clinical efficacy of anti-IL5 therapy in patients with severe eosinophilic asthma with a history of exacerbations [176,177,178], with eosinophil cut-offs set to ≥150 to ≥300 cells/μL [179] in mepolizumab trials. It has been demonstrated, however, that higher eosinophil counts than these cut-offs are associated with poor asthma control and more severe exacerbations [180]. In the study of Zieger et al. [181], a blood eosinophil count > 400 cells/μL was found to be an independent risk factor for exacerbations, emergency department visits or hospitalizations for asthma. Although blood eosinophil count levels predict the rate of exacerbations, this is not the case with sputum eosinophil count [179,182].

Total serum IgE level is a biomarker used in severe allergic asthma for the treatment with anti-IgE antibody (omalizumab). In association with elevated levels of fractional exhaled nitric oxide (FENO) (>19.5 parts per billion) and blood eosinophil count (>260/μL), it significantly predicts which patients with severe allergic asthma will respond to omalizumab, reducing the exacerbation rate [183].

The production of nitric oxide in the airways indicates Th2 type inflammation [184,185] and FeNO is a noninvasive biomarker of eosinophilic airway inflammation. There are contradictory data on whether FeNO has the ability to classify asthma severity [186,187,188]. Studies have shown that FeNO can predict accelerated decline of lung function [189], asthma relapse after corticosteroid treatment discontinuation [184], and degree of airway inflammation [190]. However, its ability to be used as a biomarker to predict exacerbations seems to be limited, even when combined with clinical features [191]. In the study by van der Valk et al. [192], repeated measurements of FENO predicted moderate asthma exacerbations (not requiring systemic corticosteroids or hospitalizations) but not severe asthma exacerbations.

Exhaled breath condensate (EBC) has been used in assessing exacerbations. Low EBC pH, various cytokines, chemokines, NO-related products, leukotrienes, and volatile organic compounds, better in combination, have been used as biomarkers associated with clinical characteristics and exacerbations [193].

Serum periostin is a biomarker of allergic eosinophilic asthma and has been used in the identification of patients who will respond to Th2-directed therapies [194]. However, limited data suggest that the serum periostin level predicts asthma exacerbations [195]. Sputum periostin, on the other hand, is associated with persistent airflow limitation, eosinophilic asthma refractory to ICS [196], while it is a potential marker for airway remodeling, as well [197,198].

There is an increasing need for developing biomarkers that will guide clinicians in the management of asthma, in terms of better and easier phenotyping asthma, predicting exacerbations, and treatment response.

## 9. Pathophysiology

Acute severe asthma commonly presents with abnormal arterial gas exchange. Arterial hypoxemia is largely attributed to ventilation/perfusion mismatch (V/Q mismatch). Hypercapnia, on the other hand, is not only present due to V/Q mismatch, but also due to respiratory muscle fatigue leading to alveolar hypoventilation. Trying to assess the exact profile of the V/Q mismatch that characterizes acute severe asthma, studies have demonstrated that although in asthma patients there is a wide spectrum of V/Q abnormalities, the most common in acute severe asthma (ASA) patients is having increased blood flow, in the context of high cardiac output, distributed in alveolar spaces with low ventilation and remarkably low V/Q ratios [199]. The pattern of ventilation-perfusion is bimodal in acute severe asthma, ranging from normally perfused areas to areas of hypoxic pulmonary vasoconstriction.

**Figure 5:**
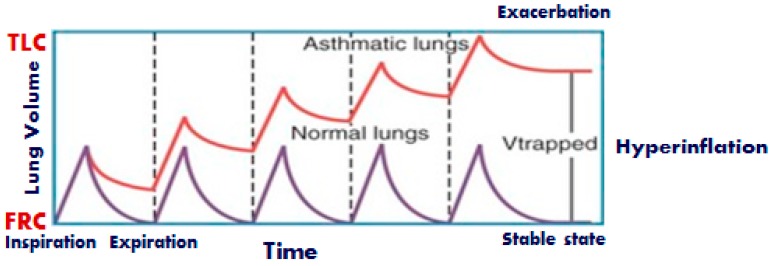
Dynamic hyperinflation during exacerbation.

With regards to the mechanics of the respiratory system, acute asthma exacerbation is characterized by reversible bronchoconstriction and increased airway resistance, followed by low flow rates, premature small airway closure, decreased elastic recoil, pulmonary hyperinflation and increased work of breathing. There is a substantial decrease in the FEV_1_ and the PEF of the patients, whereas the residual volume may increase as much as 400% of the normal and the functional residual capacity may even reach double the normal values [199]. In severe asthma exacerbations, total lung capacity (TLC) is also increasing. These changes in lung volumes help constricted airways remain open. During passive expiration of the lungs, the driving forces of the respiratory system are the elastic forces. The lower the elastic forces are, or the higher the resistive forces, the longer will the time needed to full expiration of the inspired tidal volume be, characteristic that may be quantified by a long expiratory time constant of the respiratory system. Incomplete exhalation of delivered tidal volume makes inspiration begin at a volume at which respiratory system exhibits a positive recoil pressure. The presence of flow at the end of the expiration is due to the presence of positive alveolar pressure at the end of expiration. This process is called dynamic hyperinflation and the positive end-expiratory alveolus pressure associated with higher relaxation volume is called intrinsic (auto) Positive End Expiratory Pressure (PEEP) [200] (Figure 5). Dynamic hyperinflation depends on the expiratory time constant, expiratory flow limitation, expiratory time, inspiratory muscle activity during exhalation, tidal volume, and external flow resistance [201]. Although this initially may act in favor of the patient, by reducing the resistive work of breathing, the thorax and lungs increase in volume, length–tension relationships of the respiratory muscles shorten and the strength of contraction eventually diminishes. As the severe exacerbation remains unresponsive, expiratory and accessory muscles become active, the work of breathing increases and fatigue is a serious and potentially fatal possibility, as it further compromises respiratory function and deteriorates respiratory failure. Bronchospasm and increased resistance, mucous and compression of the peripheral airways from auto-PEEP, lead to significant heterogeneity of the lung. Normal lung units coexist with pathological lung units creating a variety of many different time constants across the lung.

**Figure 6:**
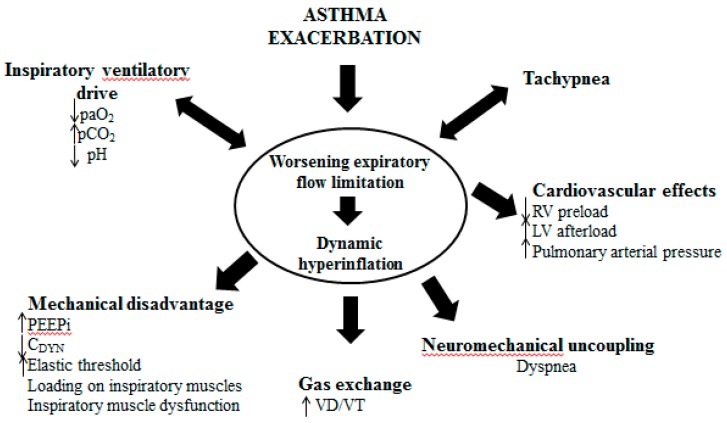
Pathophysiological changes due to dynamic hyperinflation.

Hemodynamic compromise is another important feature of a severe asthma attack that leads to significant dynamic hyperinflation. The development of positive intrathoracic pressures lead to decrease of the right heart output by decreasing right heart preload (venous return and end–diastolic volume of the right heart) and increasing right heart afterload (vascular pulmonary resistance). The decreased right heart output in parallel with the diastolic dysfunction of the left heart (caused by shifting the intraventricular septum towards the left ventricle) and its incomplete filling, lead to a significant reduction of the arterial systolic pressure in inspiration and the presence of pulsus paradoxus sign [202] (Figure 6).

Thus, due to uncontrolled or difficult to treat dynamic hyperinflation, a patient with asthma can be drowsy, confused or agitated, or may present with paradoxical thoracic-abdominal movement, with absent of wheeze in lung auscultation, bradycardia or with pulsus paradoxus. This patient is near respiratory arrest status and endotracheal intubation. Mechanical ventilation and admission to an ICU may be imminent [203].

## 10. Clinical Assessment

Identification of severe asthma exacerbations is of outmost importance, as they are related with worse outcomes and require close observation and aggressive management. A brief interview with the patient is necessary to determine certain features in the patient’s history that need to be looked into closely, because current literature identifies them as factors that increase asthma-related death. Hospital and Intensive Care Unit (ICU) admission, as well as mechanical ventilation due to an asthma exacerbation has been shown to significantly increase the risk for a new episode of near fatal and fatal asthma [204]. It is also very important to obtain a detailed description of the patient’s medication history. Medications that play a significant role in the prediction of asthma related morbidities and death are inhaled and systematic corticosteroids, as well as the use of beta agonists. In this context, not currently using inhaled corticosteroids (ICS), currently using or having recently discontinued treatment with oral corticosteroids (OCS), as well as documented overuse of short acting β agonists (SABAs) are all factors related with an increased risk for asthma associated morbidity and mortality [205,206]. Elements from the medication history may also conceal clues that may suggest inadequate treatment, or even poor adherence to a prescribed treatment plan. The lack of a written asthma plan and socioeconomic factors are also associated with a greater risk for a severe exacerbation [207].

**Figure 7:**
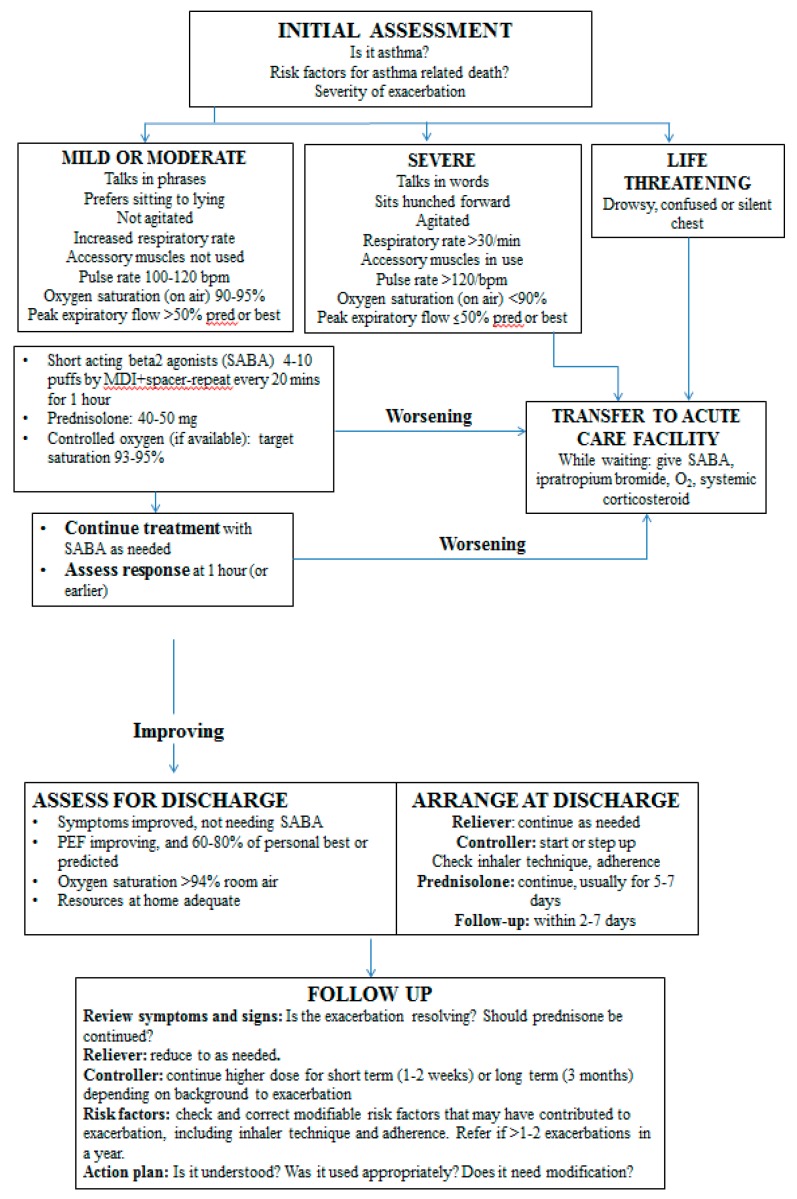
Global Initiative for Asthma (GINA) recommendations for the management of asthma exacerbations in acute care facility. PEF: Peak expiratory flow; FEV_1_: Forced expiratory volume in one second; SABA, short acting beta 2 agonists; ICU, Intensive Care Unit.

Patients suffering from an asthma exacerbation may present with a variety of signs and symptoms [208] (Figure 7). Dyspnea, chest tightness, cough and wheezing are few of those, but there is wide heterogeneity in the asthmatic patient presentation. Features that characterize acute severe asthma are agitation, drowsiness or signs of confusion, significant breathlessness at rest, with the patient talking in words, tachypnea of more than 30 breaths per minute, use of accessory respiratory muscles, tachycardia of >120 beats per minute and pulsus paradoxus. Moreover, it is crucial to identify signs that indicate an imminent respiratory arrest, such as paradoxical thoraco-abdominal movement, silent chest with absence of wheeze, bradycardia, while the absence of pulsus paradoxus might imply muscle fatigue [208]. Upon examining the patient with acute severe asthma, apart from recognizing the signs that indicate severity, it is imperative to diagnose any pathology that might attenuate the exacerbation and requires specific treatment. Such entities are pneumothorax and pneumo-mediastinum, and pneumonia. At the same time, the clinician needs to exclude conditions that may mimic the symptoms of an asthma attack, such as cardiogenic pulmonary edema, exacerbation of chronic obstructive disease, airway obstruction caused by a foreign body or an intraluminal mass, pulmonary embolism, hyperventilation syndrome and vocal cord dysfunction [209,210,211].

Although lung function measurements are less sensitive than the history of symptoms, during an asthma exacerbation, serial PEF and FEV_1_ measurements are more objective and reliable indicators of severity and should remain part of the initial assessment of an asthma patient presenting to the emergency department according to current guidelines [1,2]. Regarding PEF, the cut-off value of 50% of the patient’s best or predicted value is within the definition of an acute severe asthma episode, and requires greater attention and action. Moreover, a value of less than 33% of their best or predicted value is an indicator of life-threatening asthma. Serial monitoring of PEF may also assist the decision of either discharging the patient, should this be accompanied with a clinical improvement, or for ICU referral if the values are continuously deteriorating despite initial appropriate treatment. There is certainly a concern regarding the safety of this test in the setting of an acute exacerbation in the emergency department, and it should be performed with caution and continuous observation of the patient.

Further laboratory testing is not necessary for every patient that presents to the emergency department with an exacerbation. Chest radiographs are advised when the clinician needs to exclude conditions such as pneumonia, pneumothorax or atelectasis, but not for all patients. Arterial blood gas analysis should be performed on all patients that are critically ill, and/or are desaturating less than 92% despite treatment [212]. By performing arterial blood gas analysis, the clinician will be able to assess not only hypoxemia and the trend of PaCO_2_, but also acid base disturbances, such as respiratory acidosis and lactic acidosis which are common on acute severe asthma [213]. Further investigations may include total white blood cell count, to evaluate the potential of infection, levels of brain natriuretic peptide to exclude the presence of congestive heart failure and electrolyte level measurement.

## 11. Pharmacological and Non-Pharmacological Management

Most current guidelines regarding asthma exacerbations highlight the necessity of supplying the asthma patient with a written plan of action appropriate for their level of control, which will lead to early recognition and management of their exacerbations [1,2]. It is of outmost importance that the patients become educated on when to seek help, during the event of an acute exacerbation. In primary care and further in the emergency department or the hospital ward, a severe asthma attack needs to be identified within a short time period in order for the correct action to be taken. A severe exacerbation of asthma is a life-threatening medical emergency, thus being crucial to transfer the patient to an acute care facility, once such a condition is identified, ensuring the patient’s safety. During the transfer, it is required to provide controlled oxygen therapy, inhaled SABA, ipratropium bromide, and systemic corticosteroids. In the emergency department the pharmacological therapy of acute severe asthma should consist of SABA, ipratropium bromide, systemic corticosteroids (oral or iv), controlled oxygen therapy, and the clinician should consider the use of iv magnesium sulfate and high dose ICS [1]. (Figure 7, Table 2)

### 11.1. β2-Adrenergic Receptor Agonists

The cornerstones of acute asthma medication are bronchodilators and especially short acting beta agonists (SABA). It is recommended that in acute severe asthma SABAs are administered repetitively or continuously. These substances activate the β2 adrenoreceptors (β2ARs), which are located mainly on the smooth airway muscle cells, but are also found on other airway cells even on the inflammatory cells. Their very important characteristic is that they have a rapid onset of action, while at the same time being well tolerated, despite high doses. Although the β2 AR agonists are substances known for centuries, the great challenge remains improving their selectivity, in order to benefit from their desired effect, while at the same time reducing their adverse effects. All current asthma guidelines introduce short acting β2 agonists (SABAs), as the first line treatment for acute severe asthma. In the first steps of escalating therapy during an exacerbation, the patient is advised to increase their use “as needed”. That is also the recommendation for the primary care setting, as well as for the emergency department, where repeated inhaled administration of SABA is advised. Studies on the efficacy of nebulizers vs. metered dose inhalers (MDIs) have not proven superiority of nebulized administration. In a recent review, nebulized delivery did not improve hospital admission, length of stay in the emergency department or pulmonary function [214]. According to GINA 2018, the preferred method of administration is with strong evidence (Evidence A) pMDI with a spacer [1]. This evidence becomes less strong when referring to severe and near fatal asthma. Although continuous nebulization of SABAs was initially a very promising perspective, several studies and meta-analyses have failed to clearly demonstrate strong evidence on favor of continuous nebulized SABAs for acute asthma. Rodrigo et al. in 2002 performed a systematic review and meta-analysis that showed no difference in respiratory function measured in the first hours of administration or on the rate of hospital admissions [215]. A Cochrane systematic review on the subject, including few more studies, showed significant difference on both respiratory function and hospital admissions, in favor of the continuous use of SABA, while at the same time demonstrating a good tolerance from the patients who did not present more adverse effects with this method of administration [216]. The most commonly used SABA is salbutamol or albuterol as named in the United States, which has an onset of action of less than 10 min and duration of approximately 6 h. Lebalbuterol is a recent addition to the choices of SABAs, with its benefit of a lower than salbutamol dose that provides similar effect. There is currently evidence about its efficacy in acute severe asthma as an intermittent regimen, but not as a continuous nebulization strategy [217,218]. Continuous intravenous infusion of β2 agonists has also been proposed as a therapy, especially in patients who did not respond to intensive bronchodilation. There is no evidence to support the use of intravenous β2 agonists [219,220] or the method of continuous, subcutaneous infusions of terbutaline [221]. Epinephrine has been studied, as a nebulized, subcutaneous, intramuscular and intravenous administration, but, in current guidelines, its use is restricted for acute asthma related with anaphylaxis and angioedema [1,222,223].

### 11.2. Anticholinergics

Anticholinergic agents act as inhibitors of acetylcholine at the muscarinic cholinergic receptor. Therefore, they inhibit parasympathetic nerve impulses and they produce a beneficial effect in acute asthma, by causing airway smooth muscle relaxation. Furthermore, they enhance β2-agonist-induced bronchodilation via intracellular processes and they prolong their bronchodilator effect [61,224]. The anticholinergic agent used primarily is ipratropium bromide due to its selectivity for airway smooth muscle receptors, which reduces the systemic adverse effects. Their use is included in current guidelines for moderate to severe acute and life-threatening asthma, as well as for patients who show poor response to initial SABA therapy [1,2]. It is not recommended to use anticholinergics as a single therapy for acute asthma. It has been demonstrated that the addition of inhaled ipratropium bromide to therapy with SABAs improve hospitalization rates, relapse rates and are associated with lung function improvement [62,63,64]. This combination therapy benefit is greater for the patients who present with acute severe asthma and are at a higher risk of hospitalization. There is an increased rate of adverse effects, which are of mild nature, such as mouth dryness and tremor.

### 11.3. Corticosteroids

Within the asthma setting, it has been well established that inhaled corticosteroids reduce the rates of hospitalization and mortality for patients with asthma [65,225]. In the event of acute exacerbation, there is a different approach of their use. Current recommendations suggest that high dose ICS given within the first hour of the patient’s presentation in the emergency department, reduce the rate of hospital admissions, for patients who are not on systemic corticosteroid therapy [1]. Recent evidence however seems to be conflicting regarding their performance without the use of systemic corticosteroids, when rate of hospital admissions or changes in lung function has been studied [226,227].

Systemic corticosteroids, due to their significant anti-inflammatory properties, have a fundamental role in the management of acute asthma, and particularly for patients who present with exacerbation while receiving oral corticosteroids (OCS), or have previous history of exacerbation that required use of OCS. They are also recommended for patients who did not respond to initial SABA therapy with a prolonged effect. Apart from their role against asthma associated inflammation, they seem to increase the number and sensitivity of β-adrenergic receptors, and also restrain the migration and function of eosinophils and other inflammatory cells. On the other hand, their lack of bronchodilatory effects prohibits their use for acute asthma as a monotherapy [74]. A recent multi-center study showed that there is a significant percentage of patients who get admitted to hospital with acute asthma and do not receive systemic corticosteroids, despite the clear suggestion of current guidelines [228]. With regards to the root of administration, intravenous administration seems to not provide additional efficacy to the use of oral therapy [229,230]. Intramuscular regimens seem to be as effective as oral in reducing the risk for relapse [231]. The oral route is better tolerated and preferred, because it is quicker and less expensive. Identifying groups of patients where intravenous administration could be more beneficial is a recent field of study, and guidelines support that they should be considered for patients who may be unable to swallow due to breathlessness, or may not absorb efficiently the medication due to gastro-enteral disturbances, such as vomiting [1]. There is a lack of robust evidence to clarify the superiority of longer or higher dose OCS, thus the literature suggests a 5–7-day regimen of 50 mg prednisolone as a single dose, or 200 mg hydrocortisone in divided doses [1,2,232].

### 11.4. Magnesium Sulfate

Magnesium has been proven to be an important co-factor in enzymatic reactions and changes of its concentrations may result in different response from the smooth muscles. Hypomagnesemia may cause contraction, whereas hypermagnesemia causes relaxation of the smooth muscles and bronchodilation, possibly through inhibition of calcium influx into the muscles.

Recent recommendations include magnesium sulfate, at dose of 2 g infused over 20 min, as a second line intervention for acute severe asthma exacerbation [1,233]. It has been shown to reduce the rate of hospitalization in adults with FEV_1_ of 25–30% at presentation and those who are unresponsive to initial treatment, and have persistent hypoxemia, and correlates with improvement in lung function [234,235]. Its infusion has not been correlated with severe adverse events; it is however contra-indicated for patients with renal insufficiency, hypermagnesemia and myasthenia Gravis. Magnesium has also been tried in its nebulized form for asthma exacerbation, with very few data to support it. A recent systematic review, which examined the efficacy and safety of inhaled administration of magnesium, concluded that, although safe, it has not shown significant benefits when compared with the first line inhaled agents, thus it is not routinely recommended [236]. The current literature is reluctant to fully support the use of magnesium, mainly because of the heterogeneity of the severity of asthma attacks it has been used on in trials, especially in the context of optimized first line treatment with β2-agonists and corticosteroids [237]. A 2014 randomized controlled trial failed to show any evidence of clear benefit in the use of either intravenous or inhaled magnesium [238]. Further prospective trials are necessary to provide accurate evidence on this treatment option.

### 11.5. Methylxanthines

On the ground of their anti-inflammatory properties, methylxanthines (aminophylline and theophylline) used to be included in the primary treatment for acute asthma. Their poor safety profile, which includes significant side effects, in combination with the inability to provide evidence of improved outcomes, such as improved pulmonary function or rate of hospitalization when given for severe acute asthma, has excluded them from current guidelines [1,239]. A more recent review and meta-analysis, however, has supplied some evidence of aminophylline’s efficacy, when combined with other bronchodilators, but more data are needed on this direction [240].

### 11.6. Leukotriene Modulators

Although leukotriene receptor antagonists (LTRAs) are included as a controller agent in the asthma management, there are limited data on the efficacy of intravenous or oral antileukotriene drugs in acute asthma. Montelukast and zafirlukast were studied on patients with acute asthma and demonstrated some evidence of lung function improvement [241,242]. A review of the literature, however failed to provide robust evidence of the effectiveness of this medication category on lung function or on the outcomes of the patients [243].

### 11.7. Oxygen Supply

Although asthma exacerbations are not usually accompanied with severe hypoxemia, acute severe asthma often presents with arterial PO_2_ derangements, due to extensive *V*/*Q* mismatch as explained above. Oxygen should be administered via nasal cannula or mask, with a target of arterial oxygen saturation of 93–95%, or to those patients where saturation monitoring is not available [1]. Although not all guidelines agree on the level of the desirable target saturation, studies have shown that, in severe acute asthma, oxygen therapy with controlled low flow administration, with a target SpO_2_, is correlated with better outcomes than the use of per se high flow 100% oxygen delivery, as it has been shown to correlate with increases in PaCO_2_, as well as with decreased values of PEF [244,245]. There is also some evidence about the use of oxygen driven nebulization with SABAs, because of the pulmonary vasodilation caused by the β2-agonist, which results in increasing perfusion of poorly ventilated areas, thus resulting in deterioration of the V/Q abnormalities [246].

### 11.8. Heliox

Heliox is a mixture of helium (70–80%) with oxygen (20–30%). Heliox can be used for severe asthma exacerbations that are unresponsive to standard therapy or in patients having an upper airway obstruction component. Heliox, with density less than air, leads to lower Reynolds number, thus decreasing resistance to airflow under conditions of turbulent flow, as are prominent in the central airways and at the branch points. This effect can potentially decrease the work of breathing and improve ventilation. On the contrary, airflow in smaller airways, which are mainly affected during an asthma exacerbation, will not improve with heliox, as it is typically laminar, depending on gas viscosity rather than density.

Despite the theoretical benefits of heliox, and while a few case series have suggested a beneficial effect in acute asthma, no studies in adults have demonstrated an advantage of heliox above and beyond standard oxygen therapy. In asthma exacerbation either without or with intubation, heliox has not demonstrated consistent benefit [247,248].

Heliox has demonstrated greatest benefit for improving symptoms when used as a nebulizing gas for a beta-2 agonist medication. Benefit is generally seen within minutes after the initiation of therapy [247]. Another study has shown that using heliox as a carrier gas increase gas delivery up to 50% in a mechanical model for both MDIs and nebulizers [249]. Given that its effect is based on the percentage of helium, it should not be administrated to patients requiring FiO_2_ > 40%.

### 11.9. Ketamine

Ketamine is well known drug that has been in use since circa 1960. It is a dissociative anesthetic drug that has the potential to have different actions, depending on the dose used. It may work as a potent analgesic and as an anesthetic agent, but may also have secondary effects as a bronchodilator, while at the same time preserving airway reflexes and sympathetic nervous system tone, with no effects on the cardiovascular system. A dose of 1–2 mg/kg dose has been described as an inductive agent in rapid sequence intubation (RSI) of asthma patients [250]. In doses lower than this it does not have sedative effects, whereas in higher doses it can cause laryngospasm and apnea. Its psychoactive effects make it even less popular for use. In the context of asthma, there are no large randomized trials to examine its effect. There is some evidence of its bronchodilatory effect, especially in mild and moderate asthma exacerbations, and in doses lower than 1 mg/kg, but larger trials would be necessary to establish its role for asthma [251,252].

### 11.10. Antibiotics

There is no evidence supporting the use of antibiotics per se for severe acute asthma, unless the patient’s history and clinical assessment indicate the presence of infection. In a recent retrospective cohort study, it has been demonstrated that, in patients hospitalized with acute asthma and receiving OCS, antibiotic use was associated with longer hospital length of stay and hospital cost, whereas it held similar risk of treatment failure [253]. In a previous study in the US, 60% of the patients who were admitted to hospital with asthma exacerbation, received antibiotics, with no clear indication accompanying this decision [254]. Current guidelines suggest against their use and that they should be considered after optimizing other treatment options and when there is clear evidence of infection [1].

### 11.11. Non-Invasive Mechanical Ventilation

Although the benefits of non-invasive mechanical ventilation (NIMV) are well recognized in the acute exacerbation of chronic obstructive pulmonary disease and pulmonary edema, its usage for asthma exacerbation remains controversial. Despite the lack of supporting evidence, NIMV is commonly used in patients with severe asthma exacerbation as a mean to obviate the need for intubation and mechanical ventilation and its detrimental effects.

In the absence of clinical guidelines that recommend the use of NIMV for the management of acute asthma, evidence suggests that a trial of NIMV (for one or two hours) may be beneficial for a low risk group of patients [255], particularly those unresponsive to medical therapy. Prolonged trials of NIMV are not recommended. Suggested criteria for an NIMV trial include RR > 25 breaths per minute, heart rate > 110 beats per minute, hypoxemia with PaO_2_/FiO_2_ ratio greater than 200, hypercapnia with PaCO_2_ < 60 mmHg, FEV_1_ < 50% less than predicted and use of accessory respiratory muscles. A trial of NIMV should not be undertaken if there is any absolute criterion for endotracheal intubation (respiratory arrest, hemodynamic instability or shock, GSC < 8), excessive respiratory secretions and risk of aspiration, severe agitation and poor patient collaboration and any cause that precludes the right mask fitting (facial surgery) [256].

In a trial of 30 patients who presented to the emergency department with a severe asthma exacerbation that was not responding to inhaled bronchodilator therapy, NIMV was associated with reduction in the rate of hospitalization and increased lung function. Improvements in respiratory rate and dyspnea appear to be influenced by the amount of pressure support above expiratory positive airway pressure (EPAP) provided. The use of NIMV has been associated with reduction in endotracheal intubation, improvement in oxygenation, decrease in carbon dioxide retention, and improvement in airflow obstruction. Studies are controversial regarding the mortality and ICU length stay [86]. NIMV can also be used in the asthmatic patients who are at risk for intubation, following extubation [257].

### 11.12. Invasive Mechanical Ventilation

The decision to intubate and mechanically ventilate a near-fatal asthma patient is considered a challenging task and should be based primary on a series of clinical evaluations. Major indications for initiation of invasive mechanical ventilation (IMV) are: (1) cardiac arrest; (2) respiratory arrest or bradypnea; (3) respiratory insufficiency with PaO_2_ < 60 mmHg on 100% FiO_2_ and PaCO_2_ > 50 mmHg; (4) physical exhaustion; and (5) compromised level of consciousness. Relative indications for IMV are: (1) hypercapnia PaCO_2_ > 50 mmHg or PaCO_2_ increased by 5 mmHg per hour; (2) worsening respiratory acidosis;, (3) inability to treat patient appropriately; (4) failure to improve with proper therapy; and (5) clinical signs of deterioration and respiratory fatigue such as tachypnoea of >40 breaths per minute, severe hypoxemic respiratory insufficiency, hemodynamic instability, paradoxical thoracic movement, and silent chest [258].

The decision to intubate and mechanically ventilate a patient with acute asthma exacerbation is a clinical one and may be made urgently. When the clinician decides that respiratory failure is progressing, and is unlikely to be reversed by further pharmacologic therapy, intubation should be performed as quickly as possible by a skilled intensivist or anesthesiologist, who has extensive experience in intubation and airway management, using rapid sequence intubation (RSI) protocol suitable for asthmatic patients (good preparation, sufficient pre-oxygenation, suitable induction to anesthesia agents suitable for asthma, and placement of the endotracheal tube) [259]. Regarding the preferred method of intubation, oral endotracheal intubation is preferred, although the literature also includes awake nasotracheal intubation, which may be complicated by the fact that many asthmatic patients also have nasal polyps [203]. Additionally, oral intubation allows the use of an endotracheal tube of a larger diameter, facilitating secretion removal and bronchoscopy, if needed, while at the same time decreasing inspiratory airway resistance. It should be noted that unlike other conditions in which intubation and mechanical ventilation can solve problems, the dynamic hyperinflation that mechanical ventilation can create or even exacerbate can have devastating consequences for a severe asthmatic patient, such as cardiovascular collapse and/or barotrauma and ventilator induced lung injury. Therefore, there are certain considerations to be made before and during RSI. During RSI in such patients one should anticipate rapid oxygen desaturation despite maximal effort at pre-oxygenation especially in those patients who do not achieve a SpO_2_ above 93%, so adequate pre-oxygenation is advised. Bag mask ventilation should be done using small tidal volume and high inspiratory flow rate with a prolonged expiratory phase, attempting with this way to mimic the approach used during mechanical ventilation. Excessive mag mask ventilation should be avoided because of the risk of pneumothorax [260,261]. Manipulation of the airway can cause laryngospasm and worsening of bronchoconstriction, so one could consider the use of atropine to attenuate vagal reflexes [203]. The literature suggests the bolus use of intravenous ketamine for RSI taking advantage of its bronchodilatory effect, while propofol is also considered a safe approach. Opiates and barbiturates should be avoided due to the risk of histamine release that can exacerbate bronchoconstriction [262]. If muscle relaxants are needed, non-depolarizing muscle relaxants (except maybe atracurium and mivacurium) and succinylcholine are suitable in asthmatic patients [263].

### 11.13. Goals of Mechanical Ventilation

Near fatal asthma is characterized by severe dynamic hyperinflation of the lung with severe respiratory and circulatory consequences. The aim of mechanical ventilation is to maintain adequate oxygenation, to reduce the work of breathing and to prevent and confront further hyperinflation without any circulatory compromise or ventilator induced lung injury [264]. The intubation and post-intubation period is often complicated with severe cardio-respiratory derangement. Hypotension, the most common post-intubation complication, may be caused due to dynamic hyperinflation and auto-PEEP, and can be aggravated by dehydration, sedatives and neuromuscular blocking agents. Arrhythmias, barotraumas, laryngospasm or even seizures have also characterized the post-intubation period [265,266]. Phenomena such as hypercapnia, hypoxemia and acidemia, as well as ventilatory lung injury and life threatening pneumotrauma (pneumothorax and pneumo-mediastinum), may also complicate the post-intubation period. Reasons for the aforementioned may be the severity and non-responsiveness of the disease, but may also be the result of inadequate sedation or patient–ventilator desynchrony. Wrong and harmful initial ventilator settings may also result in providing too little or too excessive minute ventilation, potentially deteriorating the already very fragile asthmatic patient [267].

Management of the asthmatic patient post intubation starts with ensuring adequate sedation in order to achieve the desirable patient–ventilator synchronization. Sedation and analgesia will also decrease the metabolic rate, oxygen consumption and carbon dioxide production. Dexmedetominide, propofol and remifentanyl are the appropriate drugs for sedation and analgesia. Their usage has been associated with shorter length of ICU stay, shorter duration of mechanical ventilation and improved long term neurocognitive outcomes when compared to benzodiazepines [266,268]. It is important to use agents that accomplish deep sedation, while at the same time allow rapid awakening, should the patient improve quickly, which is common in the asthma cases (Table 3).

Patients with severe asthma with persistently dangerous levels of hypercapnia and arterial hypoxemia, and extreme patient–ventilatory asynchrony may require paralysis in addition to sedation. The preferred paralytic, non-depolarizing agent is cis-atracurium, as it is eliminated by esterase degradation and spontaneous breakdown in the serum. Paralytic agents can be administered either intermittently through bolus injections, or by continuous intravascular infusion. Its duration must be as short as possible, because concomitant use of intravascular corticosteroids and paralytic neuromuscular agents increases the incidence of critical illness myopathy [269,270].

**Figure 8:**
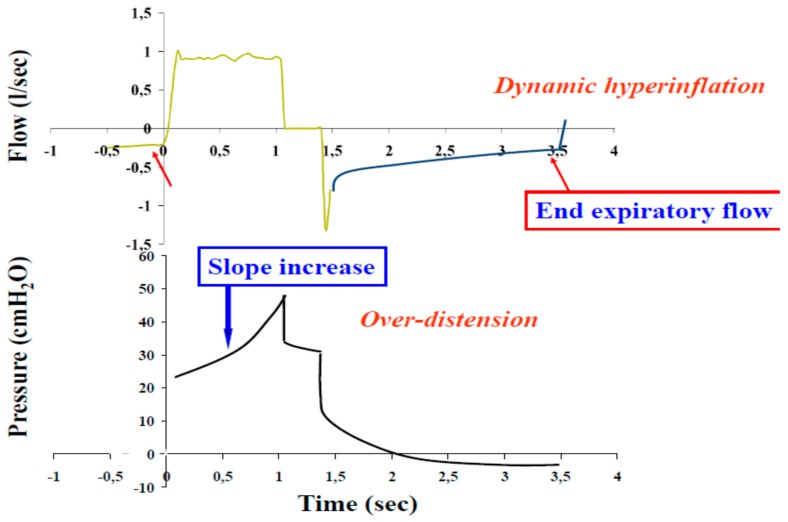
Flow time tracing of a patient with persistence of flow at the end of expiration which indicates dynamic hyperinflation and pressure time tracing with a slope increase indicative of over-distension.

To avoid the hemodynamic effects of dynamic hyperinflation, once the patient is intubated, it is advised to perform a brief discontinuation (60–90 s) from the ventilation (apnea test), a slowly bagged ventilation and to administer fluids (1–2 L or more) and vasopressors. Although there is no clear evidence to support the volume-preset over the pressure-preset modes, the preferred ventilator modes for the asthmatic patient are the volume-limited ones [263]. Barotrauma seems to occur regardless of the mode of ventilation. Volume-limited modes of ventilation are usually used for near death asthmatic patients at their entrance in the ICU. It is essential to closely monitor the Peak inspiratory and Plateau pressures, to early detect any change in resistance and compliance, and this is easily achievable when using volume modes (Figure 8). Although high inspiratory flow rates of 80 L/min up to 100 L/min and square waveforms shorten inspiratory time and increase expiration time, thus reducing hyperinflation, it has been shown that this may not have a significant impact to the degree of hyperinflation once the minute ventilation has been limited by high peak inspiratory pressure [271]. Minute ventilation should be set at a level of less than 115 mL/kg/min (less than 10 L/min) with a respiratory rate of 10–12 breaths/min and a prolonged expiratory time by decreasing I:E ratio (1:3 or 1:4 up to 1:5) [263,266]. Tuxen and Lane showed a remarkable increase in hyperinflation when using higher levels of minute ventilation [120]. The fraction of inspired oxygen (FIO_2_) should be titrated to maintain the pulse oxygen saturation (SpO_2_) above 90% (up to 94%) or the arterial oxygen tension (PaO_2_) above 60 mmHg. One should avoid SpO_2_ > 96% due to oxygen toxicity (Table 4).

Limited data exist about the use of external PEEP when ventilating a patient with severe asthma. The use of progressively higher external PEEP from 5 to 15 cmH_2_O has been shown to have a deteriorating effect both in respiratory (deterioration of the end-inspiratory volume, the functional residual capacity and plateau pressure) and circulatory system (decrease of systolic arterial pressure and cardiac output) [271]. In one prospective study of patients undergoing control mode of ventilation, external PEEP worsened hyperinflation and had serious hemodynamic effects by worsening gas trapping [272]. On the other hand, other studies have shown that the application of external PEEP, may produce a paradoxical lung deflation by reducing lung volumes and airway pressures and increasing lung homogeneity [273]. In the case of assist mode of mechanical ventilation, the application of external PEEP at a value less than 80% of the intrinsic PEEP, or 5 cm H_2_O if intrinsic PEEP is <10 cm H_2_O can counterbalance the endogenous peep and reduce the work of breathing [274]. A trial of stepwise increase in PEEP can be used and terminated when indications of worsening of dynamic hyperinflation it is shown.

### 11.14. Permissive Hypercapnia

Hypercapnia is a common fact during mechanical ventilation of asthmatic patients. PaCO_2_ levels up to 60 mmHg and pH values less than 7.20 are common on the first day of mechanical ventilation even with increased minute ventilation. The term permissive hypercapnia is a ventilating strategy that can be applied to mechanically ventilated asthma patients, that emphasizes on giving priority to the reduction of hyperinflation rather than normal minute ventilation. The reduction of minute ventilation through reduction of tidal volume and respiratory rate is used to decrease pulmonary hyperinflation. PaCO_2_ levels should rise gradually during mechanical ventilation rather than rapidly, preferably at a rate of <10 mmHg per hour or even slower if the PaCO_2_ exceeds 80 mmHg. Generally, a pH level of 7.20–7.25 is accepted, but the literature has failed to demonstrate a benefit from using alcalotic agents, such as bicarbonate infusion to accomplish that [274].

### 11.15. Additional and Unconventional Therapies for Acute Severe Asthma

#### 11.15.1. Oxygen Delivery by High Flow Nasal Canula

Oxygen delivery via high flow nasal canula (HFNC) can be used to hypoxemic patients who are not expected to respond to conventional therapies. HFNC with flow up to 60 L/min of warmed and humidified oxygen, decreases inspiratory resistance, as well as the work of breathing, can wash out carbon dioxide, thus decreasing the anatomic dead space and may also produce a positive end expiratory pressure (up to 5 mmHg) by increasing the end expiratory lung volume. The role of HFNC in asthmatic adults is unknown. Studies in children have shown that its use reduces respiratory distress in moderate and severe asthma exacerbations and also reduces the need for intubation [275,276].

#### 11.15.2. Extracorporeal Life Support (ECLS)

Extracorporeal membrane oxygenation (ECMO) is an invasive therapy, in which oxygenation and carbon dioxide removal are performed through an artificial membrane. Although evidence based on clinical trials for the use of ECMO in asthmatic patients is lacking [277,278], there is growing evidence on the subject, supporting the use of ECLS for patients receiving mechanical ventilation due to an asthmatic exacerbation. A 2009 review by Mikkelsen et al. has demonstrated that, when ECLS is used for status asthmaticus, it correlates with better outcomes in comparison to its use for other causes of respiratory failure [279]. In this study, they used data from the multicenter Extracorporeal Life Support Organization (ELSO), but included only a small number of patients. In 2017, there was another review of the same database, confirming that the use of ECMO is an acceptable option, and resulted in acceptable survival rates, although it is necessary to understand and reduce the ECMO related complications [280]. Di Lascio et al., using ECMO for asthmatic patients receiving IMV, showed that it could provide adjunctive pulmonary support for patients who remain severely acidotic and hypercapnic despite aggressive conventional therapy [281]. The writers conclude that ECMO should be considered as an early treatment in patients with status asthmaticus whose gas exchange is not satisfactory despite using conventional therapy, aiming to provide adequate gas change and to prevent ventilator induced lung injury.

A modified ECMO technique such as extracorporeal carbon dioxide removal (ECCO_2_R) may also play an important role in severe asthmatic patient in mechanical ventilation. In a difficult to safely ventilate asthmatic patient, due to extremely high airway pressures, hypoventilation and persistent severe respiratory acidemia are common issues. The usage of ECCO_2_R, considering the reversibility of the pathophysiology of asthma, provide the opportunity for more protective ventilation and more time for the bronchodilator agents to act and reverse inflammation and hyperinflation. There is no sufficient evidence to support a clear role of this technique in asthmatic patients, but there seems to be a growing interest on the subject [282,283]. Schneider et al. even presented a case where ECCO_2_R was used in an “awake” patient with a near fatal asthma attack, refractory to the use of pharmacological intervention and NIMV, resulting in avoidance of intubation [284]. However, more data are needed to establish an indication for this intervention in the context of an acute severe asthma exacerbation.

#### 11.15.3. Anesthetic Agents

Some inhalational anesthetic agents such as halothane, isoflurane and sevoflurane act as bronchodilators, probably not only through a direct relaxation effect on airway smooth muscles but also by attenuating cholinergic tone [285,286]. This characteristic may have favorable effects in patients with refractory to conventional and optimized bronchodilatory therapy. Case report studies have indicated a positive effectiveness with halothane, but also with isoflurane and sevoflurane but with several limitations. Hypotension, myocardial depression, increased ventricular irritability especially in the presence of acidosis, beta-agonists and theophylline have been reported [287,288,289]. In addition, factors such as the expense of inhalational treatment, the need of a bedside anesthesiologist, the practical issues concerning the equipment for delivering the inhalational agents, the short time of duration of bronchodilation (immediate return of bronchoconstriction after discontinuation), and, finally, the absence of randomized trials to evaluate and confirm their efficacy in near-death adult asthmatic patients make the usage of anesthetic agents a last resort as a non- conventional bronchodilatory therapy for refractory near death asthma exacerbations [290,291].

#### 11.15.4. Enoximone

Enoximone is an intravenous bronchodilatory agent that can be used in severe asthma exacerbation in adults. Enoximone, a selective phosphodiasterase inhibitor III, was tested in a study by Beute et al. on eight patients with status asthmaticus, six of whom had a respiratory arrest or hypercapnia [292]. The bronchodilatory effect was immediate. Even if the intravenous administration bypasses inhalation incapability in severe asthma, and no side-effects were observed in this study, phosphodiesterase inhibitors in general are associated with ventricular and atrial arrhythmias, hypotension, and hepatotoxicity. Further studies are needed to confirm enoximone efficacy and safety in patients with acute exacerbations of asthma that are refractory to conventional therapies.

## 12. Prognosis

Asthmatic patients who require mechanical ventilation, not only have increased hospital mortality (7%), but also long-term mortality [40,293]. Most of the long-term mortality is attributed to recurrent asthma [294]. Psychological disturbances such as depression and denial are also common features of asthmatic patients who survived a near fatal episode. Anxiety seems to be more common among close family members than the patients themselves [295]. Smoking cessation is one of the recognized factors that improves survival [151].

## 13. Prevention and Risk Reduction

**Figure 9:**
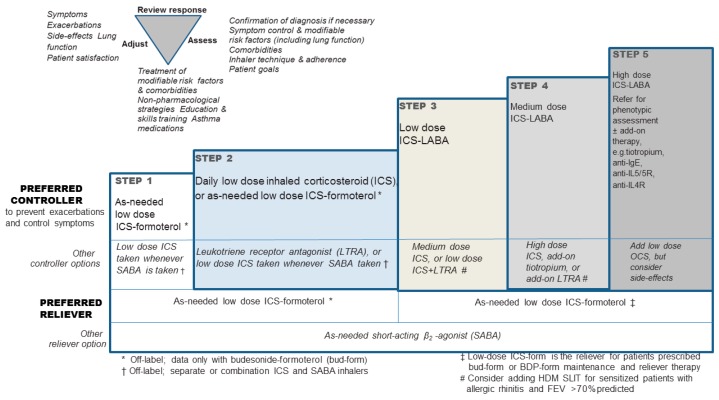
Personalized management for adults and adolescents to control symptoms and minimize future risk [1].

GINA recommends that all adults and adolescents with asthma should receive ICS-containing controller treatment, either as-needed (in mild asthma) or daily, in order to reduce their risk of serious exacerbations and to control symptoms, [1] (Figure 9). Asthma treatment should be optimized in patients continuing having poor symptom control and/or exacerbations, even though Step 4 and Step 5 treatments and contributing factors should be assessed, in order to treat modifiable risk factors that compromise disease stability (smoking, environmental exposures, allergen exposure (if sensitized on skin prick testing or specific IgE), and medications such as beta-blockers and NSAIDs) (Table 5). It is imperative to optimize the inhaler technique and adherence to treatment, as well as overuse of SABAs, and medication side effects. Furthermore, comorbidities should be assessed including obesity, GERD, chronic rhinosinusitis, obstructive sleep apnea, anxiety, depression, and social difficulties. Non-pharmacological interventions (e.g., smoking cessation, exercise, weight loss, mucus clearance, and influenza vaccination) should also be recommended where indicated.

**Figure 10:**
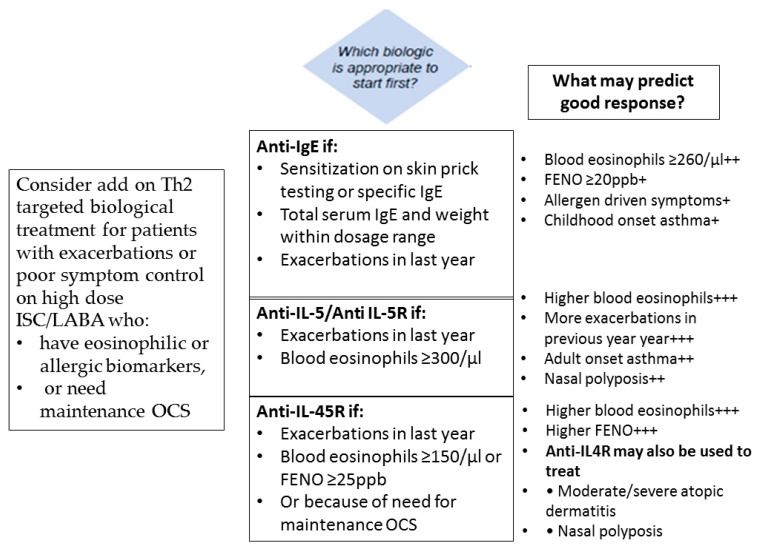
Criteria for the choice of biologic as add on treatment in Th2 driven severe asthma.

If the problems continue after having optimized all the above parameters, patients should refer to a specialist center for phenotypic assessment and consideration of add-on therapy including biologics (Figure 10). The prevalence of severe, refractory asthma is generally estimated to be 5–10% of the total asthma population [77,151]. It is important to distinguish between asthma that is difficult to control and asthma that is truly severe. Severe asthma is defined by the joint European Respiratory Society/American Thoracic Society (ERS/ATS) guidelines according to the following criteria [151]:

- Requirement for treatment with high-dose inhaled corticosteroids (ICS) and a second controller (and/or systemic corticosteroids) to maintain control.
- Refractory to the treatment mentioned above.
- Incomplete management of comorbidities such as severe sinus disease or obesity.

The GINA 2019 guidelines for adolescents and adults with difficult-to-treat and severe asthma [77] recommend that assessment of the severe asthma phenotype should be done during high dose ICS treatment (or lowest possible dose of OCS), and biological treatment should be chosen accordingly (Figure 8). Where relevant, test for parasitic infection should precede and be treated if present, before commencing Type 2 targeted treatment. The currently approved add-on biological treatments for severe asthma include anti-IgE treatment for severe allergic asthma (omalizumab), anti-IL5 or anti-IL5R for severe eosinophilic (mepolizumab, benralizumab, and reslizumab), and anti-IL4R for severe eosinophilic/Type 2 asthma or patients requiring maintenance OCS asthma (dupilumab) (Table 6).

## 14. Conclusions

Severe asthma exacerbations are a major cause of disease morbidity, functional impairment, increased healthcare costs, and increased risk of mortality. Asthma patients experience exacerbations irrespective of underlying disease severity, phenotype, or despite optimal guideline-directed treatment, as a result of the ongoing inflammatory processes and loss of the disease control. Patients with frequent emergency department visits, patients requiring hospitalization, and, more importantly, patients intubated for an asthma exacerbation are at significantly increased risk for future severe exacerbations. It is evident that prevention of exacerbations remains a major unmet need in asthma management. The identification of patients at risk to have severe exacerbations is of paramount importance. Patient education and written plans of management, control of triggering/risk factors and co-morbid conditions, monitoring of asthma control and pulmonary function as well as optimal pharmacotherapy are needed to prevent and/or decrease exacerbations. A better understanding of the pathogenesis of asthma exacerbations will ultimately lead to better strategies and the development of novel treatments in the pursuit of preventing and treating severe asthma exacerbations.

## Figures and Tables

**Table 1:** Risk factors for fatal asthma exacerbations.

A History of Near Fatal Asthma Requiring Intubation and Mechanical Ventilation
Hospitalization or emergency care visits for asthma in the past year
Currently using or having recently stopped using oral steroids
Not currently using inhaled steroids
SABA over-use (more than one canister of salbutamol/month (or equivalent))
History of psychiatric disease or psychosocial problems
Female Sex
Age > 40 years
Smoking history
Poor perception of airflow limitation
Hyperinflation in chest radiograph
Poor adherence with asthma medications and/or poor adherence
(or lack of) with a written asthma action plan
Food allergy

SABA, short acting beta agonist. Adapted from Global Initiative for Asthma (GINA) guidelines 2018 [1].

**Table 2:** Pharmacological management of patients with acute severe exacerbation in the emergency department.

Medication	Dosing	References
Salbutamol (albuterol) solution for nebulization: single dose 2.5 mg/2.5 mL	Continuous nebulization for an hour and re-assess clinical response	[214,215,216,217,218,219,220,221,223]
Ipratropium bromide	Nebulization of 0.5 mg/2.5 mL/4–6 h in combination with salbutamol (same nebulizer)	[61,62,63,224]
Corticosteroids	Methylprednisolone iv infusion of 40 mg or hydrocortisone iv, 200 mg or oral prednisone 40 mg	[65,225,226,227,228,229,230,231,232]
Magnesium sulfate	Single iv infusion of 2 gr/20 min	[233,234,235,236,237,238]
Methylxanthines	Not recommended as first line; poor response and potential serious adverse events	[239,240]
Leukotriene receptor antagonists	Single iv infusion of 7–14 mg over 5 min	[241,242,243]
Epinephrine (adrenaline)	0.3–0.4 mL sc of a 1:1000 (1 mg/mL) solution/20 min for 3 doses in case of no response	[222]
Terbutaline (1 mg/mL)	0.25 mg sc/20 min for 3 doses in case of no response (preferred in pregnancy)	[221,223]
Heliox	Helium/oxygen mixture in a 80:20 or 70:30 ratio	[247,248,249]

iv, intravenous; sc, subcutaneous.

**Table 3:** Sedation, analgesia and paralysis in patients with acute severe asthma exacerbation requiring intubation.

Medication	Dosing	Side Effects	References
Midazolam	0.03–0.1 mg/kg bolus iv infusion, followed by an infusion of 3–10 mg/h	Hypotension	[268,269]
Propofol	Infusion of 60–80 mg/min initially, up to 2 mg/kg. Continue with iv infusion of 5–10 mg/kg/h as needed, and for sedation on mechanical ventilation 1–4 mg/kg/h	Hypotension, seizures, hyperlipidemia	[262,268]
Fentanyl	50–100 μg/kg bolus iv infusion, followed by infusion of 50–100 μg/h	Bradycardia, histamine release	[268,269]
Remifentanyl	Initial dose of 1 μg/kg iv infusion, followed by an infusion of 0.25–0.5 μg/kg/min (up to 2 μg/kg/min)	Bradycardia, hypotension	[268,269]
Ketamine	1 mg/mL bolus iv infusion, followed by a maintenance infusion of 0.1–0.5 mg/min	Sympatheticomimetic effects, delirium	[250,251,252]
Dexmedetomidine	Initial loading dose of 1 μg/kg, iv over 10–30 min, followed by a maintenance infusion of 0.2–0.7 μg/kg/h	Hypotension, bradycardia	[68,269]
Cis-atracurium	0.1–0.2 mg/kg bolus iv infusion, followed by infusion in a rate of 3 μg/kg/min (up to 10 μg/mL/min)	Bronchospasm	[269,270]

**Table 4:** Initial ventilator settings in intubated patients with acute severe asthma exacerbation.

Mode	Settings
Tidal volume	6 mL/kg ideal bodyweight
Respiratory rate	8–10/min
Minute ventilation	<10 L/min
Inspiratory flow rate	60–80 L/min
Inspiratory to expiratory ratio	>1:3
Inspiratory wave form	Decelerated waveform
Expiratory time	4–5 s
Plateau pressure	<30 cm H_2_O
PEEP	0 cm H_2_O
FiO_2_	100% initially and titrate to maintain SaO_2_ > 90%

SaO_2_: Oxygen saturation; Peep: positive end expiratory pressure.

**Table 5:** Modifiable risk factors that have to be treated in order to reduce exacerbations.

Risk Factor	Treatment Strategy	Evidence
Any patient with 1 risk factor for exacerbations (including poor symptom control)	Ensure patient is prescribed an ICS-containing controller	A
	Ensure patient has a written action plan appropriate for their health literacy	A
	Review patient more frequently than low-risk patients	A
	Check inhaler technique and adherence frequently	A
≥1 severe exacerbation in last year	Consider alternative controller regimens to reduce exacerbation risk, e.g., ICS-formoterol maintenance and reliever regimen	A
	Consider stepping up treatment if no modifiable risk factors	A
	Identify any avoidable triggers for exacerbations	C
Exposure to tobacco smoke	Encourage smoking cessation by patient/family; provide advice and resources	A
	Consider higher dose of ICS if asthma poorly-controlled	B
Low FEV_1_, especially if <60% predicted	Consider trial of 3 months of treatment with high-dose ICS and/or 2 weeks of OCS	B
	Exclude other lung disease, e.g., COPD	D
	Refer for expert advice if no improvement	D
Obesity	Strategies for weight reduction	B
	Distinguish asthma symptoms from symptoms due to deconditioning, mechanical restriction, and/or sleep apnoea	D
Major psychological problems	Arrange mental health assessment	D
	Help patient to distinguish between symptoms of anxiety and asthma; provide advice about management of panic attacks	D
Major socioeconomic problems	Identify most cost-effective ICS-based regimen	D
Confirmed food allergy	Appropriate food avoidance; injectable epinephrine	A
Allergen exposure if sensitized	Consider trial of simple avoidance strategies; consider cost	C
	Consider step up of controller treatment	D
	Consider adding SLIT in symptomatic adult HDM-sensitive patients with allergic rhinitis despite ICS, provided FEV1 is >70% predicted	B
Allergen exposure if sensitized	Increase ICS dose independent of level of symptom control	A

FEV_1_, forced expiratory volume in 1 s; HDM, house dust mite; ICS, inhaled corticosteroids; OCS, oral corticosteroids; SLIT, sublingual immunotherapy.

**Table 6:** Currently available biologics: indications and adverse effects.

Medication	Use	Adverse Effects
Anti-IgE (omalizumab, SC, ≥6 years)	An add-on option for patients with severe allergic asthma uncontrolled on high dose ICS-LABA. elf-administration may be permitted	Reactions at the site of injection are common but minor. Anaphylaxis is rare.
Anti-IL5/anti-IL5R (anti-IL5 mepolizumab (SC, ≥12 or ≥6 years), reslizumab (IV, ≥18 years) or anti-IL5 receptor benralizumab (SC, ≥12 years))	Add-on options for patients with severe eosinophilic asthma uncontrolled on high dose ICS-LABA	Headache and reactions at injection site are common but minor.
Anti-IL4R (dupilumab, SC, ≥12 years)	An add-on option for patients with severe eosinophilic/Type 2 asthma uncontrolled on high dose ICS-LABA, or requiring maintenance OCS. It is also approved for treatment of moderate-severe atopic dermatitis. Self-administration may be permitted	Reactions at injection site are common but minor. Blood eosinophilia occurs in 4–13% of patients.
